# Ten quick tips for teaching programming

**DOI:** 10.1371/journal.pcbi.1006023

**Published:** 2018-04-05

**Authors:** Neil C. C. Brown, Greg Wilson

**Affiliations:** 1 Department of Informatics, King's College London, London, United Kingdom; 2 DataCamp, Toronto, Ontario, Canada; Genome Quebec, CANADA

This is a *PLOS Computational Biology* Education paper.

## Introduction

Research from educational psychology suggests that teaching and learning are subject-specific activities [1]: learning programming has a different set of challenges and techniques than learning physics or learning to read and write. Computing is a younger discipline than mathematics, physics, or biology, and while there have been correspondingly fewer studies of how best to teach it, there is a growing body of evidence about what works and what doesn't. This paper presents 10 quick tips that should be the foundation of any teaching of programming, whether formal or informal.

These tips will be useful to anyone teaching programming at any level and to any audience. A larger list aimed primarily at K–12 audiences can be found at [2].

## Tip 1: Remember that there is no geek gene

Guzdial [3] refers to the belief that some people are born programmers and others aren't as "computing's most enduring and damaging myth." This is often "confirmed" by looking at university grade distributions, which are commonly held to be bimodal: a low-scoring hump of those who will never get it and a high-scoring hump of those who have the right stuff. Our first and most important tip is that this is wrong: competence at programming is not innate but is rather a learned skill that can be acquired and improved with practice.

The most powerful evidence for this comes from Patitsas et al. [4]. They examined grade distributions in introductory computing courses at a large university and found that only 5.8% were actually multimodal. More damningly, they found that computer science faculty were more likely to see distributions as bimodal if they thought those grades came from a programming class than if they believed the grades came from some other kind of class and that those faculty were even more likely to see the distributions as bimodal if they believed that some students are innately predisposed to do well in computer science.

Beliefs such as this are known to have powerful effects on education outcomes [5–7]. If instructors believe that "some kids get it, and some kids don't", they will (consciously or unconsciously) invest less in those whom they put in the second category. When combined with cultural stereotypes about who is and isn't a "natural programmer," the downward spiral of underachievement that results from differential attention may be partly responsible for the gender imbalance in computing.

## Tip 2: Use peer instruction

One-on-one tutoring is perhaps the ideal form of teaching: all of a teacher's attention can be focused on one student, and they can completely customise their teaching for that person and tailor individual feedback and corrections based on a two-way dialogue with them. In realistic settings, however, one teacher must usually teach several, tens, or even hundreds of students at once. How can teachers possibly hope to clear up many learners' different misconceptions in these larger settings in a reasonable time?

The best method developed so far for larger-scale classrooms is called peer instruction. Originally created by Eric Mazur at Harvard [8], it has been studied extensively in a wide variety of contexts, including programming [9, 10]. In simplified form, peer instruction proceeds in several phases:

1. The instructor gives learners a brief introduction to the topic.

2. The instructor then gives learners a multiple choice question that probes for misconceptions rather than simple factual recall. (A programming example is given in Code 1 that relates to integer comparison and loops.) The multiple choice question must be well designed. There is no point asking a trivial question that all students will get right or one with meaningless wrong answers that no student will pick. The ideal questions are those for which 40%–60% of students are likely to get the right answer the first time ([11], p. 23) and those in which every wrong answer corresponds to a misconception that will cause it to be picked by at least some students.

3. Learners then vote on the answer to the question individually, thus formalising their initial prediction.

4. Next, learners are given several minutes to discuss those answers with one another in small groups (typically 2–4 students), and they then reconvene and vote again.

5. Then, the instructor can act on the latest answers. If all the learners have the right answer, the instructor can move on. If some of the wrong answers remain popular after group discussion, the instructor can address those specific misconceptions directly or engage in class-wide discussion.

Peer instruction is essentially a way to provide one-to-one mentorship in a scalable way. Group discussion significantly improves learners' understanding because it forces them to clarify their thinking, which can be enough to call out gaps in reasoning. Repolling the class then lets the instructor know if they can move on or if further explanation is necessary. While it significantly outperforms lecture-based instruction in most situations, it can be problematic if ability levels differ widely (as they often do in introductory programming classes because of varied prior experience). Pair programming (Tip 5) can be used to mitigate this.

## Tip 3: Use live coding

Rather than using slides, instructors should create programs in front of their learners [12]. This is more effective for multiple reasons:

1. It enables instructors to be more responsive to "what if?" questions. While a slide deck is like a highway, live coding allows instructors to go off-road and follow their learners' interests or answer unanticipated questions.

2. It facilitates unintended knowledge transfer: students learn more than the instructor consciously intends to teach by watching how instructors do things. The extra knowledge may be high level (e.g., whether a program is written top-down or bottom-up) or fairly low level (e.g., learning useful editor shortcuts).

3. It slows the instructor down: if the instructor has to type in the program as they go along, they can only go twice as fast as their learners, rather than 10-fold faster as they could with slides—which risks leaving everyone behind.

4. Learners get to see how instructors diagnose and correct mistakes. Novices are going to spend most of their time doing this, but it's left out of most textbooks.

5. Watching instructors make mistakes shows learners that it's alright to make mistakes of their own [13]. Most people model the behaviour of their teachers: if the instructor isn't embarrassed about making and talking about mistakes, learners will be more comfortable doing so too.

Live coding does have some drawbacks, but with practice, these can be avoided or worked around:

1. Instructors can go too slowly, either because they are not good typists or by spending too much time looking at notes to try to remember what they meant to type.

2. Instructors can spend too much time typing in boilerplate code that is needed by the lesson but not directly relevant to it (such as library import statements). Not only does this slow things down, it can distract learners from the intended thrust of a lesson. As Willingham [14] says, "Memory is the residue of thought"; if the instructor spends their time typing boilerplate, that may be all that learners take away. This can be avoided by starting with a partial skeleton that includes the boilerplate or having it on hand to copy and paste when needed. (Of the two, we prefer the former, since learners may not be able to keep up with copying and pasting.)

Note that live coding does not always have to start with a blank screen: instructors may give students some starter code that relies solely on concepts they have already mastered and then extend it or modify it with live coding. Instructors who use live coding should ensure that learners have reference material available after lectures, such as a textbook, but should also recognize that students of all ages increasingly turn to question and answer sites such as Stack Overflow for information.

## Tip 4: Have students make predictions

When instructors are using live coding, they usually run the program several times during its development to show what it does. Surprising research from peer instruction in physics education shows that learners who observe a demonstration do not learn better than those who did not see the demonstration [15], and in fact, many learners misremember the outcome of demonstrations afterwards [16]. In other words, demonstrations can actually be useless or actively harmful.

The key to making demonstrations more effective is to make learners predict the outcome of the demonstration before performing it. Crucially, their prediction should be in some way recorded or public, e.g., by a show of hands, by holding up cue cards marked with A, B, C, or D, or by talking to their neighbour. We speculate that the sting of being publicly wrong leads learners to pay more attention and to reflect on what they are learning; regardless of whether this hypothesis is true, instructors should be careful not to punish or criticise students who predicted wrongly but rather to use those incorrect predictions as a spur to further exploration and explanation.

## Tip 5: Use pair programming

Pair programming is a software development practice in which 2 programmers share 1 computer. One person (called the driver) does the typing, while the other (called the navigator) offers comments and suggestions. The two switch roles several times per hour. Pair programming is a good practice in real-life programming [17] and also a good way to teach [18]. Partners can not only help each other out during practical exercises but can also clarify each other's misconceptions when the solution is presented.

Both parties involved in pair programming learn while doing it. The weaker gets individual instruction from the stronger, while the stronger learns by explaining and by being forced to reconsider things that they may not have thought about in a while. When pair programming is used, it is important to put everyone in pairs, not just the learners who may be struggling, so that no one feels singled out. It's also important to have people switch roles within each pair 3 or 4 times per hour so that the stronger personality in each pair does not dominate the session.

## Tip 6: Use worked examples with labelled subgoals

Learning to program involves learning the syntax and semantics of a programming language but also involves learning how to construct programs. A good way to guide students through constructing programs is the use of worked examples: step-by-step guides showing how to solve an existing problem.

Instructors usually provide many similar programming examples for learners to practice on. But since learners are novices, they may not see the similarity between examples: finding the highest rainfall from a list of numbers and finding the first surname alphabetically from a list of names may seem like quite different problems to learners, even though more advanced programmers would recognise them as isomorphic.

Margulieux and Morrison et al. [19–21] have shown that students perform better when worked examples are broken down into steps (or subgoals) that are given names (or labels)—an example is given in Code 2. Subgoal labels provide a structure that allows learners to see the similarities between coding problems and to communicate with their peers and instructors more efficiently. Learners can then apply the labels to future tasks that they attempt themselves.

## Tip 7: Stick to one language

A principle that applies across all areas of education is that transference only comes with mastery [22]. Courses should therefore stick to one language until learners have progressed far enough with it to be able to distinguish the forest from the trees. While an experienced programmer can, for example, take what they know about loops and function calls in one language and reuse that understanding in a language with a different syntax or semantics, a newcomer does not yet know which elements of their knowledge are central and which are accidental. Attempting to force transference too early—e.g., requiring them to switch from Python to JavaScript in order to do a web programming course early in their education—will confuse learners and erode their confidence.

## Tip 8: Use authentic tasks

Guzdial et al. found that having learners manipulate images, audio, and video in their early programming assignments increased retention in 2 senses: learners remembered more of the material when retested after a delay and were more likely to stay in computing programs [23]. This is a particular instance of a larger observation: learners find authentic tasks more engaging than abstracted examples.

A classic question in computing (and mathematics) education is whether problems are better with context (e.g., find the highest student grade) or without (e.g., find the maximum of a list of numbers). Bouvier et al. [24] examined this with a multiuniversity study and found no difference between the two. They suggest that since it makes no difference, other considerations (such as motivation) should be given priority.

One caution about choosing context is that context can inadvertently exclude some people while drawing others in. For example, many educators use computer games as a motivating example for programming classes, but some learners may associate them with violence and racial or gender stereotypes or simply find them unenjoyable. Whatever examples are chosen, the goal must be to move learners as quickly as possible from "hard and boring" to "easy and exciting" [25].

To help students accomplish a visible and satisfying result quickly, instructors can provide some prewritten software libraries or source code that starts students closer to the end goal. The idea that students must start from scratch and write all the code they need themselves is the relic of a bygone era of home microcomputers (and it was not true even then). Pick the task that you actually want to the students to engage in and provide everything else premade.

## Tip 9: Remember that novices are not experts

This principle is tautological, but it is easily forgotten. Novices program differently than experts [26] and need different approaches or tools. If you ask a professional programmer to iterate over a list of integers and produce the average, they can write the code within seconds, using stored knowledge of the exact pattern required. Novices will approach this problem totally differently: they need to remember the syntax for the different parts, know how to iterate over a list, know how to use an accumulator variable, and so on.

Novices may need to spend time thinking about an algorithm on paper (something expert programmers rarely need, as they have usually memorised most common algorithmic patterns). They may need to construct examples in guided steps. They may struggle to debug. Debugging usually involves contrasting what is happening to what should be happening, but a novice's grasp on what should be happening is usually fragile.

Novices do not become professionals simply by doing what professionals do at a slower pace. We do not teach reading by taking a classic novel and simply proceeding more slowly. We teach by using shorter books with simpler words and larger print. So in programming, we must take care to use small, self-contained tasks at a level suitable for novices, with tools that suit their needs and without scoffing.

## Tip 10: Don't just code

Our final tip for teaching programming is that you don't have to program to do it. Faced with the challenges of learning syntax, semantics, algorithms, and design, examples that seem small to instructors can still easily overwhelm novices. Breaking the problem down into smaller single-concept pieces can reduce the cognitive load to something manageable.

For example, a growing number of educators are including Parsons Problems in their pedagogic repertoire [20, 27]. Rather than writing programs from scratch, learners are given the lines of code they need to solve a problem, but in jumbled order. Reordering them to solve the problem correctly allows them to concentrate on mastering control flow without having to devote mental energy to recalling syntax or the specifics of library functions. They are also liked by learners; Ericson et al. [28] found that learners were more likely to attempt Parsons Problems than nearby multiple choice questions in an e-book.

## Conclusion

The 10 tips presented here are backed up by scientific research. Like any research involving human participants, studies of computing education must necessarily be hedged with qualifiers. However, we do know a great deal and are learning more each year. Venues like SIGCSE (Technical Symposium on Computer Science Education, http://sigcse.org/), ITiCSE (Conference on Innovation and Technology in Computer Science Education, http://iticse.acm.org/), and ICER (International Computing Education Research Conference, https://icer.hosting.acm.org) present a growing number of rigorous, insightful studies with immediate practical application. Future work may overturn or qualify some of our 10 tips, but they form a solid basis for any educational effort to the best of our current knowledge.

We offer one final observation: do not forget the human element. Programmers have a reputation for pouring scorn on certain programming tools (e.g., pouring scorn on spreadsheets) or for gatekeeping (e.g., stating that you cannot learn programming if you did not start young). If you are teaching someone to program, the last thing you want to do is make them feel like they can't succeed or that any existing skill they have (no matter when or how acquired) is worthless. Make your learners feel that they can be a programmer, and they just might become one.

**Code 1. An example multiple choice question probing learners' understanding of loops and integer comparisons**

for (int i = 1; i < 10; i++) {

    if (i < 3 || i > = 8) {

        System.out.println("Yes");

    }

}

How many times will the above code print out the word ‘Yes’?

    a) 10

    b) 5

    c) 4

    d) 3

**Code 2. An example of subgoal labelling**

    **Conventional Materials**

    1. Click on "AccelerometerSensor1"

    2. Drag out a when AccelerometerSensor1.AccelerationChanged block

    3. Click on "cowbellSound"

    4. Drag out call cowbellSound. Play and connect it after when AccelerometerSensor1.AccelerationChanged

    **Subgoal-Labelled Materials**

    Handle Events from My Blocks

    1. Click on "AccelerometerSensor1"

    2. Drag out a when AccelerometerSensor1.AccelerationChanged block

    Set Output from My Blocks

    3. Click on "cowbellSound"

    4. Drag out call cowbellSound. Play and connect it after when AccelerometerSensor1.AccelerationChanged
